# Cell Wall Remodeling and pH Stress Coordinately Regulate Monascus Pigment Biosynthesis Through Transcriptional Reprogramming

**DOI:** 10.3390/foods14213602

**Published:** 2025-10-23

**Authors:** Xufeng Wang, Hailei Zhao, Chengfang Ding, Wentao Ding, Qingbin Guo, Changlu Wang

**Affiliations:** State Key Laboratory of Food Nutrition and Safety, School of Food Science and Engineering, Tianjin University of Science & Technology, No. 9, 13th Street, Tianjin Economic and Technological Development Area, Tianjin 300457, China

**Keywords:** monascus pigments, cell wall architecture, pH stress, transcriptomics, biosynthesis regulation

## Abstract

Monascus pigments (MPs), natural food colorants produced by *Monascus* spp., have been traditionally used in China and Southeast Asia. Our prior work demonstrated that altered cell wall architecture in *M. purpureus* M9 significantly enhances pigment synthesis and secretion, although biosynthetic regulation under combined cell wall stress and acidic conditions remains unexplored. This study employed comparative transcriptomics to investigate coordinated regulation of MP production by pH stress and modified cell wall polysaccharides in wild-type (M9-WT) and UDP-galactopyranose mutase-deficient (M9-KO) strains at pH 5.0 and 3.0. At pH 5.0, *MpglfA* knockout enhanced MP secretion through cell wall restructuring involving differential expression total 67 genes (DEGs) of primary metabolism. Acidic stress (pH 3.0) significantly increased DEGs (168 up/643 down) in M9-KO versus M9-WT, inducing amino acid/fatty acid degradation pathways that generate MP precursors (acetyl-CoA/propionyl-CoA) and accelerating metabolic transition toward secondary metabolism. Concurrently, M9-KO adopted survival strategies featuring growth suppression and acid stress pathway activation to coordinate osmotic adaptation. Glucan synthase genes exhibited greater pH sensitivity than galactomannan-related genes, while MP biosynthetic genes were transcriptionally repressed in M9-KO under higher acidity. KEGG (Kyoto Encyclopedia of Genes and Genomes) enrichment and the series test of cluster confirmed that primary metabolic pathways, particularly nitrogen/carbon metabolism, critically regulate MP biosynthesis. Transcriptomic analysis under limited pH regimes revealed that antagonistic regulators ROX1 and SPT15 mediated pH-responsive transcriptional reprogramming, potentially regulating specific MP biosynthesis (e.g., monascus orange pigments). This work established theoretical foundations for manipulating cell wall composition to enhance MP production efficiency.

## 1. Introduction

Monascus pigments (MPs), a class of azaphilone compounds produced by *Monascus* spp., are categorized into three major types based on absorption wavelengths: monascus red pigments (MRPs), monascus orange pigments (MOPs), and monascus yellow pigments (MYPs). Within the food industry, MPs serve primarily as natural color additives. The characteristic hue of MRP enables the direct application of mixed pigments as red colorants, eliminating the need for separation and purification steps. Beyond coloring, MPs exhibit diverse bioactivities including anti-inflammatory, antioxidant, antibacterial, cholesterol-lowering, antimutagenic, and antitumor effects, extending their utility to cosmetics and textile industries [1]. Recent studies identify specific MYP components (Monascin and Ankaflavin) as agents capable of reducing serum cholesterol, triglycerides, and low-density lipoprotein cholesterol (LDL-C), while elevating high-density lipoprotein cholesterol (HDL-C) levels, thereby contributing to blood pressure reduction. MYP has also demonstrated antitumor, antibacterial, anti-obesity, and anti-inflammatory properties, revealing significant market potential in functional foods and nutraceuticals [2].

In contrast, research and industrial application of MOP remain limited in food and cosmetics, primarily due to low yields and challenges in purification. To date, reports of industrial-scale MOP production are scarce. Consequently, enhancing the yield of individual MPs represents a critical challenge in current monascus pigment production. Advances in modern molecular biology and omics technologies provide powerful tools for investigating MP metabolic regulation. Omics, the study of relationships among biological components, adopts a holistic approach to analyze cellular structures, genes, proteins, and their interactions, capturing the global state of genes, RNA, proteins, and metabolites within a system for comprehensive biological interpretation [3]. Transcriptomics stands as the most widely and successfully applied omics technique in monascus metabolic studies. For instance, transcriptomic analyses, alone or integrated with other omics, elucidated regulatory mechanisms governing MP synthesis in response to blue light [4], static magnetic fields [5], and alterations in cell membrane/wall composition [6], accelerating research progress in this field.

Current evidence links MP synthesis to glycolysis, pyruvate metabolism, the TCA cycle, and amino acid metabolism. Metabolomic studies indicate close associations between MRP synthesis and intracellular amino acids, while MOP and MYP correlate with nucleotide metabolism [7]. This suggests coordinated regulation of MP types at metabolomic and transcriptomic levels, complicating targeted metabolic engineering for individual MPs.

Phosphate limitation suppresses MRP production [8], a process that demonstrates particular sensitivity to nitrogen restriction. Under nitrogen limitation, MRP synthesis is significantly repressed with accompanying proteomic changes spanning amino acid biosynthesis, protein translation, antioxidant enzymes, glycolysis, and transcriptional regulators. This metabolic reorganization reflects a flux shift from glycolysis toward the TCA cycle to maintain cellular energy homeostasis at the expense of the polyketide biosynthesis pathway [9].

Nitrogen source composition critically influences pigment distribution. NH_4_Cl or NH_4_NO_3_ as the sole nitrogen source significantly increases the proportion of MOP and MRP, whereas nitrate specifically promotes hydrophilic MYP biosynthesis [10]. Integrated omics analyses reveal that this MYP promotion involves upregulated biosynthetic genes coupled with enhanced expression of proteins, including non-ribosomal peptide synthase, oxidoreductase, glucoamylase, endo-1,4-β-xylanase, O-acetylhomoserine sulfhydrylase, and isocitrate lyase. Concurrent activation of ergosterol biosynthesis, membrane transport, and secretion pathways [11] provides essential precursors and facilitates transmembrane transport of these hydrophilic pigments. Further regulation occurs through the pentose phosphate pathway, which transcriptionally controls MYP biosynthesis [12].

However, achieving high yields of lipophilic MOP via submerged fermentation proves exceptionally difficult. Consequently, most current research targets water-soluble MYP. The aqueous environment of submerged fermentation impedes dispersion of lipophilic MOP into the extracellular milieu. Additionally, MOP acts as a precursor for the non-enzymatic conversion to other MPs (e.g., MRP), further reducing its accumulation. Our group unexpectedly discovered that a UDP-galactopyranose mutase-deficient strain exhibits dramatically enhanced pigment secretion capacity, particularly a substantial increase in extracellular MOP yield, highlighting its potential for lipophilic extracellular MOP production. Nevertheless, the regulatory mechanism underlying high-level MOP synthesis remains unclear.

Therefore, this study employs pH, a key environmental factor critically influencing monascus secondary metabolite yield, to investigate metabolic differences between the wild-type strain M9-WT and the knockout strain M9-KO under varying pH conditions. The aim is to elucidate the metabolic regulatory mechanisms governing MPs and exopolysaccharide synthesis/secretion based on altered cell wall structure and the molecular mechanisms underpinning pH-dependent metabolic adaptation to osmotic stress.

## 2. Materials and Methods

### 2.1. Strains and Culture Condition of Spore

The UDP-galactopyranose mutase-deficient *Monascus* strain (M9-KO) was derived from the wild-type *Monascus purpureus* M9 (M9-WT). Both strains were inoculated onto CYA slant agar medium containing the following per 100 mL: FeSO_4_·7H_2_O (0.001 g), K_2_HPO_4_ (0.1 g), MgSO_4_·7H_2_O (0.05 g), KCl (0.05 g), NaNO_3_ (0.3 g), sucrose (3 g), yeast extract (0.5 g), and agar powder (1.5 g). The pH was unadjusted, and the medium was sterilized at 121 °C for 20 min. Following sterilization, strains underwent 10-day cultivation at 30 °C to promote sporulation. Spore suspensions were subsequently obtained by washing the culture surfaces with sterile water and filtered through a 40 μm sterile cell strainer to remove hyphae. The resulting filtered spore suspensions were collected for subsequent use.

### 2.2. Fermentation and Pretreatment of the Mycelium

Two grouping factors were selected, strain type (M9-WT or M9-KO) and pH (pH 3.0 or pH 5.0), and cross-combined to establish four experimental groups: M9-WT at pH 5.0, M9-KO at pH 5.0, M9-WT at pH 3.0, and M9-KO at pH 3.0. Prepared spore suspensions of both strains were individually inoculated into fermentation medium, achieving a final spore concentration of 1 × 10^4^ spores/mL for submerged fermentation. The fermentation medium contained the following per 100 mL: tryptone (2 g), NaNO_3_ (1 g), K_2_HPO_4_ (1.0 g), NH_4_Cl (0.5 g), MgSO_4_·7H_2_O (0.5 g), and fructose (6 g). The initial pH of the medium was adjusted using HCl (10%, *V*/*V*) conditionally. The fermentation was conducted at 30 °C with agitation at 200 rpm for 6 days. Following fermentation, cultures were harvested and filtered through Buchner funnels lined with nylon membranes (with a pore size of 10 μm) to collect mycelia. Excess surface moisture was removed from the mycelia using sterile filter paper. Precisely 2.0 g aliquots of mycelia were weighed into 5 mL centrifuge tubes, flash-frozen by immersion in liquid nitrogen for 5 min, and subsequently transferred to −70 °C storage for preservation.

### 2.3. RNA-Sequencing and Analysis

#### 2.3.1. Sequencing Data Quality Control and Assessment

Construction of cDNA libraries and transcriptome sequencing were outsourced to MajorBio Corp. (Shanghai, China). Paired-end sequencing was performed on the Illumina HiSeq 2500 platform with the read length of 2 × 150 bp. The average sequencing depth was 3000×, and the libraries were constructed using a non-strand-specific protocol. Raw sequences underwent quality control and statistical processing using Fastp (version 0.19.5; https://github.com/OpenGene/fastp (accessed on 26 May 2024)), which executed the following procedures: removal of adapter sequences from reads, trimming of low-quality bases from read termini, discarding reads containing >5 consecutive N bases, and exclusion of reads with <30 bp length post-trimming. Following quality control, re-statistics and quality assessment were performed on the processed data.

#### 2.3.2. Reference Genome Alignment and Functional Annotation of Transcripts

Clean reads were aligned to the reference genome using HISAT2 (version 2.1.0; http://ccb.jhu.edu/software/hisat2/index.shtml (accessed on 26 May 2024)), generating mapped reads. These mapped reads were subsequently used for transcript assembly and expression quantification. The *Monascus purpureus* HQ1 reference genome (assembly accession: GCA_006542485.1; https://www.ncbi.nlm.nih.gov/assembly/GCA_006542485.1 (accessed on 26 May 2024)) was retrieved from the NCBI Genome Database. Following the reference genome alignment of transcripts, assembled sequences underwent comprehensive structural and functional annotation using the following databases: NR (NCBI Non-redundant Protein Sequences; ftp://ftp.ncbi.nlm.nih.gov/blast/db/ (accessed on 1 June 2024)), GO (Gene Ontology; http://www.geneontology.org (accessed on 1 June 2024)), KEGG (http://www.genome.jp/kegg/ (accessed on 1 June 2024)), Swiss-Prot (Curated Protein Sequence Database; http://web.expasy.org/docs/swiss-prot_guideline.html (accessed on 1 June 2024)), eggNOG (Evolutionary Genealogy of Genes: Non-supervised Orthologous Groups; http://www.ncbi.nlm.nih.gov/COG/ (accessed on 1 June 2024)), and Pfam (Protein Family Database; http://pfam.xfam.org/ (accessed on 1 June 2024)).

#### 2.3.3. Transcript Quantification and Differential Expression Analysis

Transcript quantification was performed using RSEM (version 1.3.3; http://deweylab.biostat.wisc.edu/rsem/ (accessed on 9 October 2024)) to determine global expression levels of genes/transcripts. Differential expression analysis was subsequently conducted across samples using DEGseq. Following quantification of gene/transcript read counts, DEGseq enabled the identification of differentially expressed genes (DEGs) or transcripts between sample groups. DEGs were defined using thresholds of |log_2_FC| ≥ 1 and adjusted *p*-value < 0.05.

#### 2.3.4. Enrichment Analysis of DEGs

Functional enrichment analysis was performed on target gene/transcript sets to elucidate associated biological functions and key metabolic pathways. Enrichment analyses encompassed both GO term enrichment and KEGG pathway enrichment, as well as series test of cluster. The analysis was conducted using hierarchical clustering with the following parameters: the distance metric was Euclidean distance, and the linkage method was average linkage. The number of clusters was pre-set to 10 based on the study’s objective to capture major expression trends while maintaining interpretability. Gene clustering and sample clustering were both performed using this hierarchical approach with average linkage. The expression matrix used for clustering was generated using RSEM software (version 1.3.3) with FPKM as the quantification metric. Prior to clustering, expression values were log10-transformed to reduce the influence of extreme values. Genes with total expression sums below 1 across all samples were excluded from the analysis.

#### 2.3.5. Determination of Gene Expression by qRT-PCR

Candidate regulatory genes identified through differential transcript expression screening underwent RT-qPCR validation to confirm their functional roles in polysaccharide and pigment biosynthesis. Total RNA was isolated from mycelia harvested following 6-day cultivation. Immediate reverse transcription generated cDNA libraries. Amplification kinetics and fluorescence signals were monitored using a Stratagene Mx3000P qPCR system (Agilent Technologies (Santa Clara, CA, USA)). Relative gene expression quantification followed established methodology [13].

## 3. Results

### 3.1. Gene Expression Profiling

**Figure 1:**
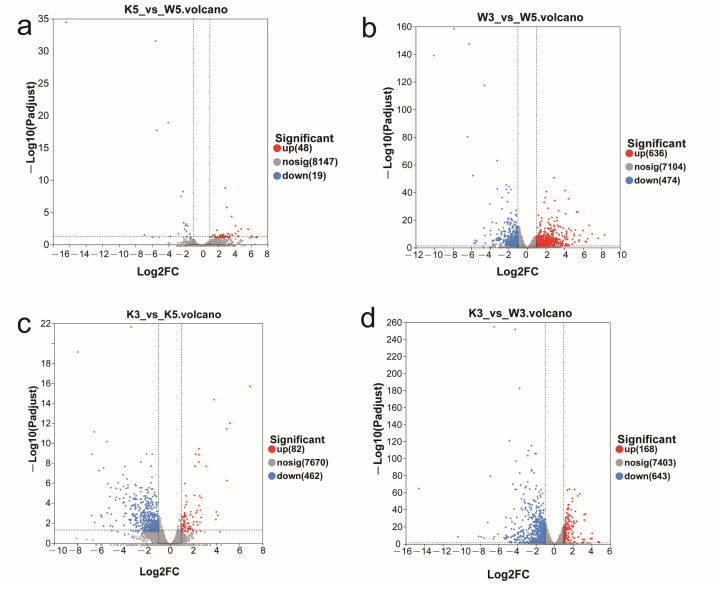
The volcano maps of differentially expressed genes among different samples. Sample comparisons were designated as follows: k5 vs. w5 (**a**), w3 vs. w5 (**b**), k3 vs. k5 (**c**), and k3 vs. w3 (**d**). The dashed lines demarcate the thresholds for statistically significant differential expression (|log2FC| ≥ 1 and adjusted *p*-value < 0.05).

**Figure 2:**
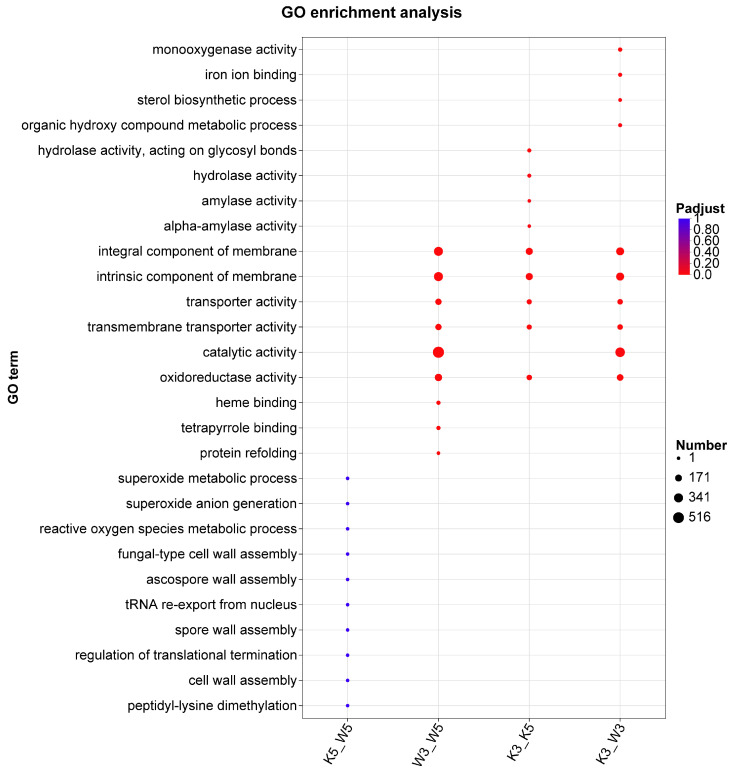
GO clustering analysis of differentially expressed genes.

Comprehensive sequencing metrics—including data quality, genome alignment efficiency, and transcriptome-wide expression distributions—are documented in Supplementary Tables S1–S3, confirming dataset integrity for downstream analysis. Fragments per kilobase of exon model per million mapped fragments (FPKM values, the predominant expression quantification method [14]) revealed high transcriptional activity across most genes (FPKM > 1 defines expressed genes; Table S3), although substantial inter-sample heterogeneity was evident (Figure 1). Hierarchical DEG distribution across comparison groups followed this descending order: w3 vs. w5 (greatest divergence) > k3 vs. w3 > k3 vs. k5 > k5 vs. w5 (minimal divergence). Crucially, wild-type (M9-WT) and knockout (M9-KO) strains exhibited fundamentally distinct pH response signatures: (1) under standard cultivation (pH 5.0), M9-KO vs. M9-WT showed negligible transcriptional divergence (67 DEGs: 48 up/19 down; Figure 1a); (2) acid stress (pH 3.0) induced markedly more DEGs in both strains versus their pH 5.0 baselines, yet with opposing regulatory polarity—wild-type displayed upregulation dominance (636 up/474 down; Figure 1b), whereas knockout exhibited predominant downregulation (82 up/462 down; Figure 1c); and (3) critically, M9-KO vs. M9-WT comparisons at pH 3.0 generated dramatically amplified differential expression (168 up/643 down; Figure 1d), overwhelmingly skewed toward downregulation.

Collectively, these results demonstrate that *MpglfA* deletion exerts marginal effects on global transcription under physiological conditions. However, its ablation profoundly amplifies acid stress responsiveness, manifesting as extensive transcriptional repression that starkly contrasts with wild-type adaptive upregulation. This establishes *MpglfA* as a critical modulator of pH-dependent gene regulatory networks.

### 3.2. GO Enrichment Analysis of Differentially Expressed Genes

GO enrichment analysis of differentially expressed genes (DEGs) was performed to elucidate biological functions and metabolic processes underlying inter-sample variations (Figure 2). At pH 5.0, DEGs between knockout and wild-type strains (k5_w5) were primarily associated with superoxide metabolic processes, superoxide anion generation, reactive oxygen species metabolic, fungal-type cell wall assembly, regulation of translational termination, cell wall assembly, and peptidyl-lysine demethylation. These findings indicate that *MpglfA* deletion primarily affects cell wall biogenesis and oxidative processes, potentially explaining altered cell wall polysaccharide composition in the mutant strain.

Under acidic conditions (pH 3.0), DEGs encompassed broader biological processes with higher gene counts compared with pH 5.0. Key activities included redox metabolism (monooxygenase and oxidoreductase activities), membrane restructuring (sterol biosynthetic process and membrane constituent components), and metabolite transport (transporter and transmembrane transporter activities). This demonstrates heightened sensitivity of the knockout strain to acidic stress.

Intrastrain comparisons revealed distinct metabolic adaptations. Wild-type strain DEGs (w3_w5) were enriched for membrane components, transporter activities, oxidoreductase activity, catalytic activity, heme binding, tetrapyrrole binding, and protein refolding. The knockout strain (k3_k5) additionally showed enrichment in hydrolyzing O-glycosyl compounds, amylase activity, and α-amylase activity, suggesting coordinated regulation of carbon source hydrolysis to optimize resource allocation under stress.

Notably, membrane constituents, transporter activities, and oxidoreductase activity were highly enriched in three experimental groups (k3_w3, w3_w5, and k3_k5) but not in k5_w5, indicating that reduced pH predominantly affects membrane biogenesis, biosynthesis, and transport processes.

### 3.3. KEGG Pathway Enrichment Analysis of DEGs

**Figure 3:**
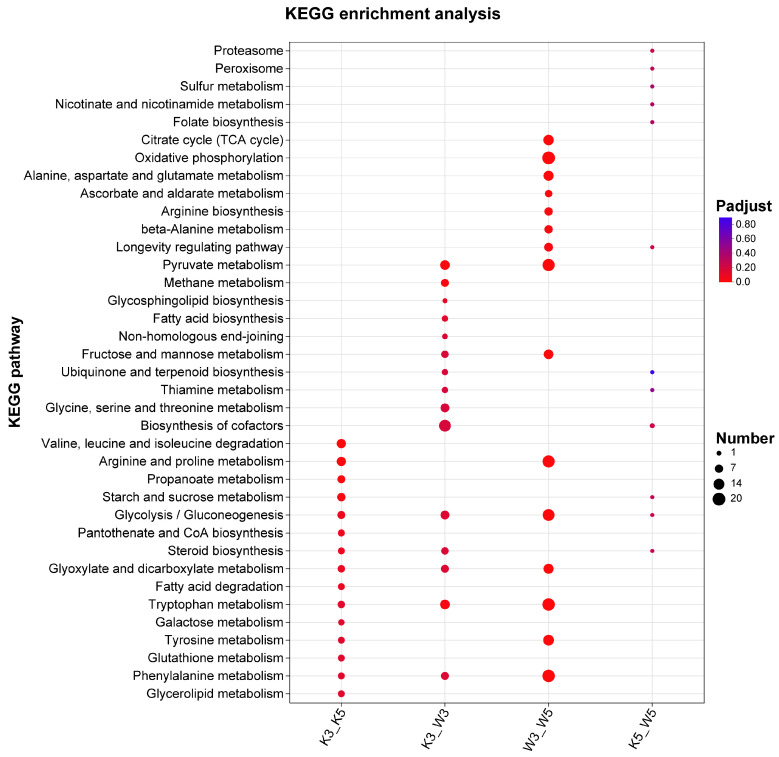
KEGG clustering analysis of differentially expressed genes.

KEGG enrichment analysis of strain-specific differential genes under varying pH conditions revealed distinct metabolic adaptations (Figure 3). At pH 5.0, the M9-KO vs. M9-WT comparison (k5_w5) implicated limited metabolic alterations, primarily involving proteasome, peroxisome, sulfur metabolism, and cofactor biosynthesis pathways—all characterized by modest gene counts and subtle expression changes. Strikingly, acid stress (pH 3.0) dramatically expanded the metabolic divergence in k3_w3, engaging pathways including pyruvate metabolism, glycerolipid metabolism, fatty acid degradation, and non-homologous end joining. Significantly enriched processes absent in wild-type comparisons encompassed sphingolipid biosynthesis, terpenoid-quinone synthesis, and thiamine metabolism—all critical for stress resilience. Glycerolipid metabolism stabilize membranes under acid stress [15], and fatty acids and terpenoids maintain fluidity [16,17]. Thiamine further regulates stress-responsive gene expression and signaling [18,19], while cofactor biosynthesis enrichment suggests pH-responsive signal transduction activation. These adaptations indicate that the knockout strain mounts a comprehensive stress response at pH 3.0, prioritizing osmotic balance through specialized metabolite synthesis while suppressing growth-related activities. Those were consistent with compromised cell wall integrity diminishing environmental resilience.

Cross-pH comparisons further illuminated strain-specific strategies. Wild-type (w3_w5) maintained core energy pathways (TCA cycle and oxidative phosphorylation) and amino acid metabolism. Conversely, the knockout (k3_k5) displayed fundamental metabolic restructuring: depletion of central carbon metabolism (TCA cycle, glycolysis) coincided with enhanced valine/leucine/isoleucine degradation and fatty acid β-oxidation—generating acetyl-CoA and propionyl-CoA precursors that fuel secondary metabolism like MP biosynthesis [20]. This metabolic shift occurred alongside significantly reduced enrichment breadth and magnitude in k3_k5 versus w3_w5. Crucially, downregulated genes vastly outnumbered upregulated counterparts in the knockout at pH 3.0 (Figure 1c), demonstrating acid-induced growth suppression alongside accelerated entry into secondary metabolism, which constitutes a compensatory survival mechanism for structural vulnerabilities.

### 3.4. Regulatory Impact of pH on Polysaccharide Biosynthesis

**Figure 4:**
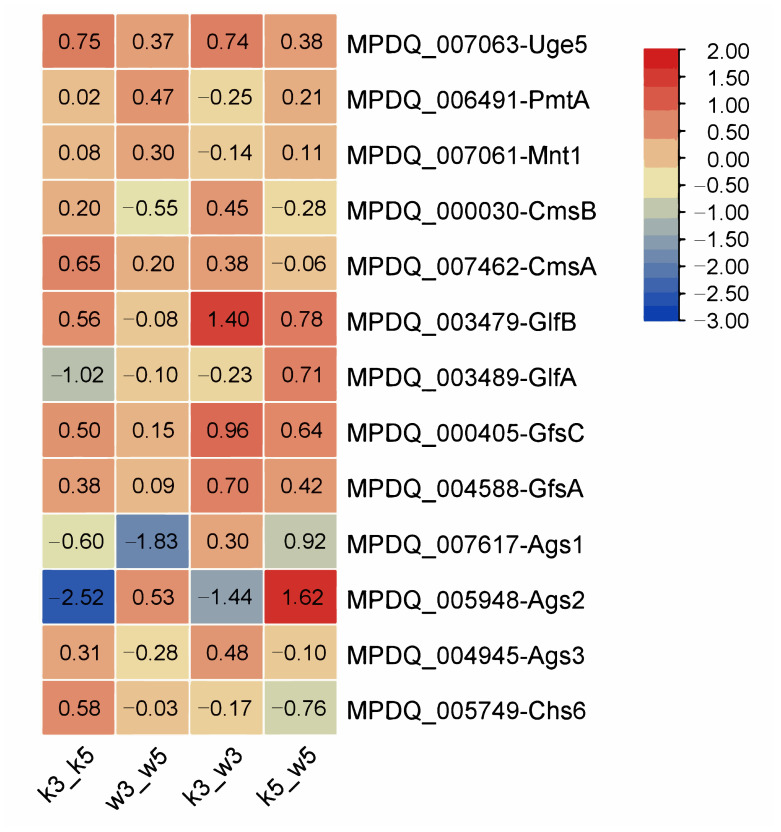
Distribution of differential expression multiples of polysaccharide synthesis genes among samples in *Monascus*.

In our prior investigation, genomic bioinformatic analysis of *Monascus* spp. identified three principal polysaccharide classes: galactomannan, glucan, and chitin [21]. To investigate pH-dependent polysaccharide biosynthesis, we profiled expression trends of key synthetic genes across four sample groups. Targeted genes included galactomannan pathway components (*uge5*, *pmtA*, *mnt1*, *cmsB*, *cmsA*, *glfB*, *MpglfA*, *gfsC*, and *gfsA*), predicted glucan synthases (*ags1–3*) [22], and chitin synthase *chs6* [23]. Expression significance was defined by |log_2_FC| > 1, with all values visualized in Figure 4.

At pH 5.0, no significant differential expression occurred in any polysaccharide synthesis genes between M9-KO and M9-WT (k5_w5), consistent with minimal yield variations observed in prior studies [21]. Similar expression stability under acidic stress indicated negligible pH influence on galactomannan regulation. However, k3_w3 exhibited significant upregulation of *glfB* (1.40-fold), which mediates UDP-galactofuranose transport to the Golgi apparatus [24]. Trace galactose detection in extracellular polysaccharides suggests *glfB* overexpression may facilitate galactosyl incorporation, although the full UDP-galactofuranose biosynthesis pathway requires further validation. Intrastrain comparisons revealed stronger pH-induced disruption of galactomannan synthesis genes in M9-KO (k3_k5) versus M9-WT (w3_w5), with the interference effect further amplified in interstrain acid-stressed comparisons (k3_w3).

Glucan synthase genes displayed divergent regulation patterns. In k5_w5, *ags1* and *ags3* were downregulated, while *ags2* was upregulated; this polarity reversed in k3_w3 (*ags1*/*ags3* upregulated, *ags2* downregulated). Parallel trends emerged in intrastrain comparisons, potentially reflecting structural and spatial roles of glucans in cell wall architecture. Crucially, glucan synthases demonstrated greater pH sensitivity than galactomannan-related genes. Chitin synthase *chs6* exhibited minimal responsiveness to acid perturbation across all comparisons.

### 3.5. pH-Dependent Modulation of Pigments Biosynthesis

**Figure 5:**
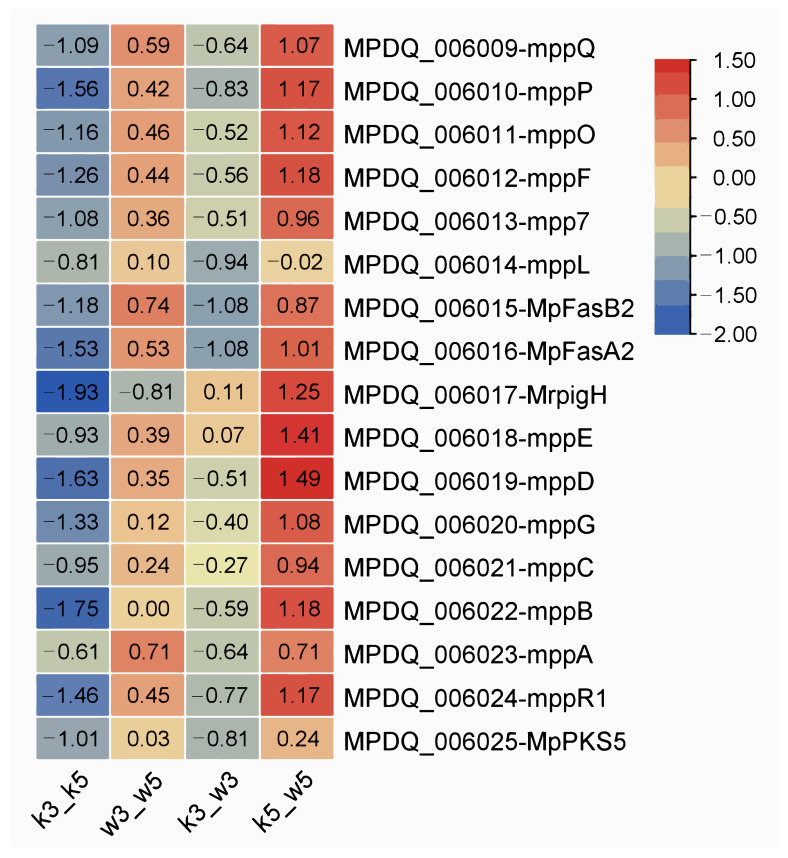
Distribution of differential expression multiples of MP genes among samples in *Monascus*.

There are over 16 genes within the monascus pigment (MP) biosynthetic cluster [20]. We examined expression patterns of key characterized genes—including *mppQ*, *mppP*, *mppO*, *mppF*, *mpp7*, *mppL*, *MpFasB2*, *MpFasA2*, *MrpigH* (homologous to *pigH*), *mppE*, *mppD*, *mppG*, *mppB*, *mppA*, and *mppR1*—across four sample groups using comparative transcriptomics. Differential expression (|log_2_FC|) distributions are shown in Figure 5.

Under standard conditions (pH 5.0), M9-KO versus M9-WT (k5_w5) exhibited modest upregulation of most MP genes, potentially reflecting early fermentation sampling where rapid growth precedes substantial pigment accumulation. Conversely, acidic stress (pH 3.0) dramatically suppressed nearly all MP genes in k3_w3 except *MrpigH* and *mppE*. This indicates severe impairment of MP biosynthesis in the knockout strain under extreme acidity.

Intrastrain comparisons revealed divergent pH responses: M9-KO (k3_k5) showed non-significant downregulation trends across MP genes, while M9-WT (w3_w5) maintained slight upregulation. Collectively, pH perturbation exerted minimal influence on MP synthesis within individual strains. However, interstrain comparison under acid stress demonstrated significant transcriptional repression specifically in the knockout strain, highlighting its heightened vulnerability to acidic conditions.

### 3.6. Series Test of Cluster of MP Biosynthesis-Associated DEGs

**Figure 6:**
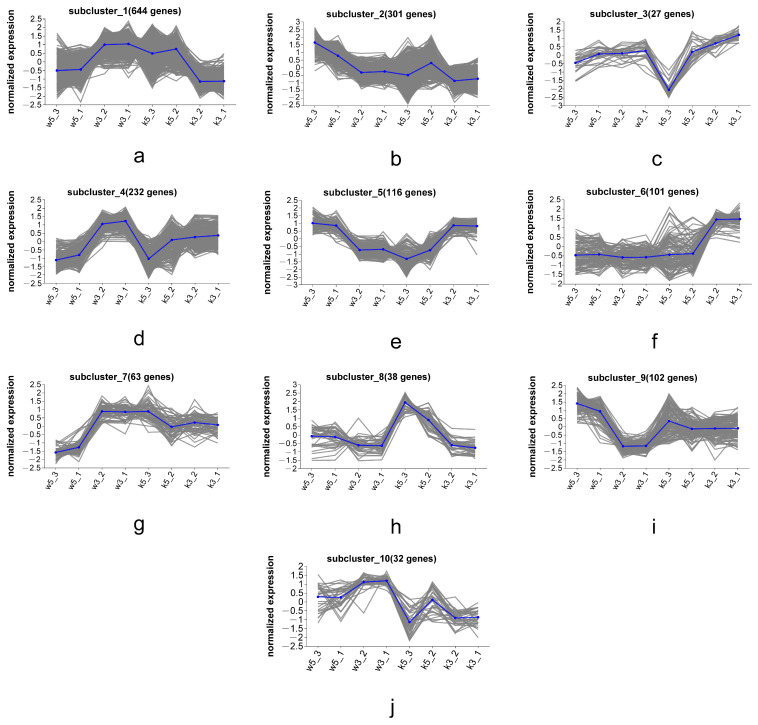
The subclustering classification (**a**–**j**) of differentially expressed genes among different transcripts.

To elucidate the metabolic regulation of MP secretion by cell wall mechanisms, we profiled expression patterns of MP-associated genes and transcripts across samples, performed co-expression clustering based on transcriptional profiles, and identified functionally relevant genes governing MP biosynthesis/secretion through expression pattern similarity analysis. Hierarchical clustering of global transcriptomes (w5, w3, k5, and k3) revealed 10 distinct expression subclusters (Figure 6), with each subcluster exhibiting characteristic expression patterns across conditions.

**Figure 7:**
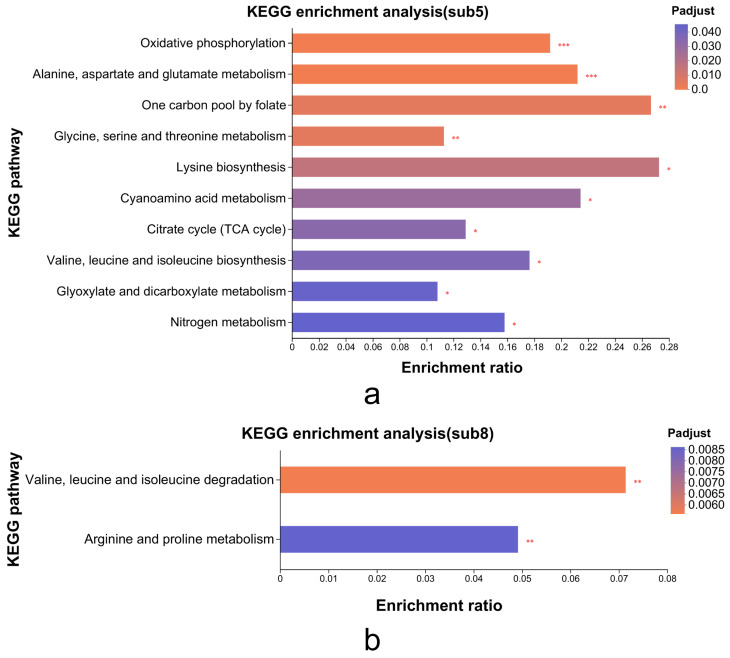
Analysis of metabolic pathways significantly enriched with differentially expressed genes in subcluster_5 (**a**) and subcluster_8 (**b**). (* *p* < 0.05, ** *p* < 0.01, *** *p* < 0.001), representing significantly enriched pathways.

Subcluster_5 (Figure 6e) and subcluster_8 (Figure 6h) displayed expression profiles positively and negatively correlated, respectively, with MP biosynthetic gene patterns (Figure 5). Consequently, we conducted KEGG enrichment analysis on these subclusters (116 DEGs in subcluster_5, 38 DEGs in subcluster_8) to identify metabolic pathways linked to MP synthesis/secretion. Subcluster_5 genes significantly enriched oxidative phosphorylation, TCA cycle, and amino acid metabolism pathways (alanine/aspartate/glutamate, glycine/serine/threonine, and lysine biosynthesis; Figure 7). Subcluster_8 primarily enriched branched-chain amino acid degradation (valine/leucine/isoleucine) and arginine/proline metabolism.

KEGG analysis confirmed critical involvement of primary metabolic processes in MP synthesis, especially nitrogen/carbon metabolism through amino acid biosynthesis/degradation and the TCA cycle. These interconnected pathways serve dual roles: (1) supplying carbon skeletons and energy for growth and (2) generating secondary metabolite precursors like acetyl-CoA and propionyl-CoA. Valine/leucine/isoleucine degradation (Supplementary Figure S1) exemplifies this metabolic mechanism, where upregulated expression of MPDQ_004788 (2-oxoisovalerate dehydrogenase β-subunit), MPDQ_003394 (isovaleryl-CoA dehydrogenase), and MPDQ_003395 (methylcrotonyl-CoA carboxylase) enhances branched-chain amino acid catabolism. This process generates acetyl-CoA and propionyl-CoA derivatives that serve as essential precursors for de novo MP biosynthesis.

Oxidative phosphorylation further supports MP biosynthesis by providing essential energy and redox equivalents. As Supplementary Table S4, subcluster_5 contained multiple genes encoding ATP synthase components (e.g., ATP1 [25], ATP20 [26], and ATP9 [27]) and respiratory chain elements (CYC1_1 [28], QCR2 [29], COX5 [30], KGD2 [31], and IDH1 [32]), whose coordinated upregulation ensures adequate energy supply for metabolic demands, including pigment production.

### 3.7. Regulatory Factor Profiling in pH Response Mechanisms

Integrating functional annotations with transcriptional profiles revealed 14 highly expressed candidate transcription factors (TFs; Supplementary Table S5). These TFs participate in fungal transcriptional initiation (SPT15, CRE1, SFP1, and HAP2), nitrogen metabolism (GAT1 and GZF1), cellular differentiation (RLM1), and stress/adaptive responses (MSN1, RST2, RFX1, ROX1, YOX1, MCM1, and CAT8). Specifically, MPDQ_005936 (MSN1), MPDQ_005046 (RFX1), and MPDQ_007404 (ROX1) were implicated in osmotic stress regulation. MSN1 activates invertase/glucoamylase expression while mediating invasive growth and osmotic stress responses [33]. RFX1 bidirectionally modulates target genes based on cellular conditions [34]. ROX1 transcriptionally regulates sterol biosynthesis pathways [35], commonly leveraged for metabolic engineering of yeast sterol metabolism.

**Figure 8:**
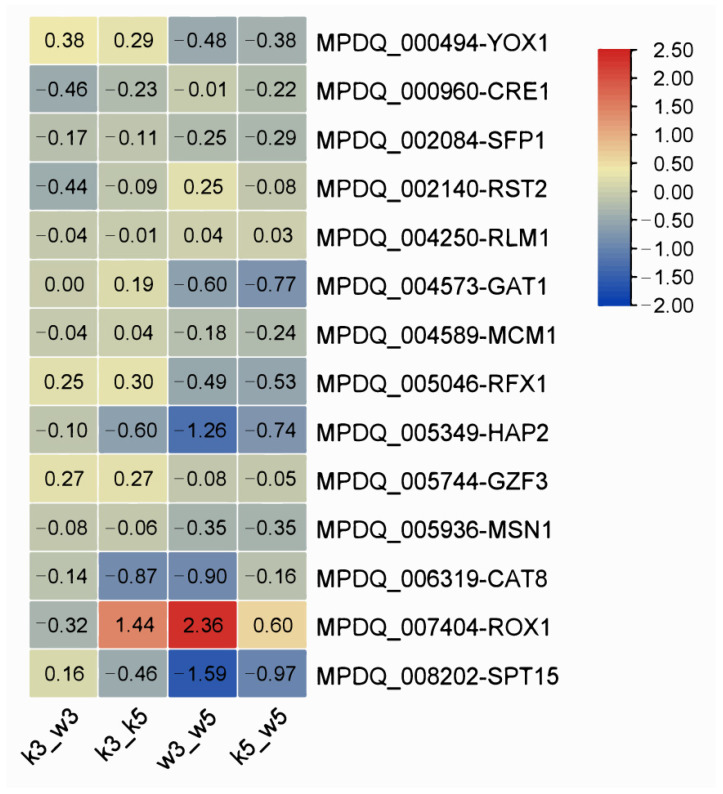
Distribution of differential expression multiples of transcriptional regulatory factors among samples in *Monascus*.

Expression patterns across comparison groups (Figure 8) identified significant differential regulation of MPDQ_008202 (SPT15), MPDQ_007404 (ROX1), and MPDQ_005349 (HAP2) in w3_w5. SPT15 was downregulated in w3_w5, k3_k5, and k5_w5 but slightly upregulated in the inter-strain acid-stressed comparison (k3_w3), suggesting its role as a global pH-responsive regulator. Conversely, ROX1 exhibited inverse regulation where it was upregulated in w3_w5, k3_k5, and k5_w5 but downregulated in k3_w3. This complementary expression profile implies SPT15 and ROX1 may function as antagonistic master regulators coordinating acid stress adaptation.

## 4. Discussion

This study investigates how alterations in monascus cell wall polysaccharide architecture and osmotic stress stimulation mechanistically regulate MP biosynthesis. Current models posit that increased precursor availability (e.g., acetyl-CoA/propionyl-CoA) elevates MP production [36], while external stimuli like blue light or static magnetic fields activate MAPK signaling to induce pigment synthesis gene cluster (PSGC) expression [4,5]. Nevertheless, direct transcriptional regulators of PSGC remain unidentified. Fungal cell wall integrity (CWI) pathways detect structural anomalies of the cell wall and initiate compensatory responses. In *S. cerevisiae*, sensor Rho1p activates Pkc1p-MAPK cascades, culminating in transcription factor Rlm1p regulating wall biogenesis genes [37]. RlmA, homologous to Rlm1p, governs chitin synthases (*chsA-G*, *csmB*), α-glucan synthase (*ags1-3*), and β-1,3-glucan synthase (*fks1*) expression in aspergilli [38]. In this work, putative glucan synthase *Mpags2* showed 1.6-fold higher expression in M9-KO versus M9-WT, potentially explaining differential polysaccharide synthesis observed previously [21]. These findings implicate CWI pathways in cell wall remodeling-mediated MP secretion, although insignificant *Rlm1* differential expression in M9-KO precludes definitive confirmation of its regulatory role in MP synthesis.

A further investigation into the metabolic mechanisms governing MOP synthesis in Monascus revealed that nitrogen metabolism and nucleotide metabolism are closely linked to MOP production [7]. Our study yielded parallel observations, confirming significant involvement of amino acid metabolism and nitrogen metabolic pathways in MP biosynthesis (Figure 7). The literature consistently reported a strong correlation between MOP and MRP production, limiting their separate yields—a phenomenon closely associated with the specific forms of amino nitrogen sources. Furthermore, current research on MOP remains largely confined to intracellular studies due to the considerable difficulty in extracellular secretion of liposoluble MOP. Prior work established that altered cell wall architecture enhances extracellular MP yield and PSGC expression. We thus propose extracellular MP secretion critically drives sustained synthesis. MP production typically stabilizes upon reaching intracellular accumulation thresholds due to feedback inhibition—a universal biological phenomenon (e.g., in yeast, homocitrate synthase catalyzes L-lysine biosynthesis but is strongly inhibited by the end product [39]). However, continuous MP efflux from structurally compromised cells maintains sub-threshold intracellular levels, delaying feedback suppression and enabling prolonged synthesis with elevated extracellular pigment yields, particularly for MOP. Enhanced secretion of intracellular MOP substantially reduces its potential conversion into other pigments, leading to pronounced extracellular accumulation. Although extracellular MOP may theoretically convert to MRP in the presence of amino groups and their derivatives, our prior studies demonstrated maintained high MOP proportions. This phenomenon may be attributed to our use of nitrate nitrogen rather than ammonium nitrogen [7], which restricts MRP synthesis. The resulting high MOP concentration thereby further elevates the extracellular MOP/MRP ratio.

**Figure 9:**
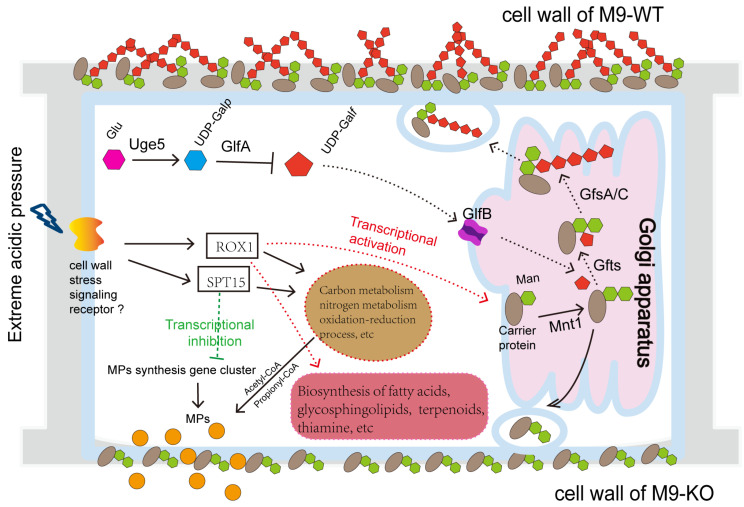
Schematic diagram of the metabolism patterns for MP synthesis and secretion in M9-KO under an extreme acidic environment. The key components of the biosynthesis pathway of *O*-mannose type galactomannan include enzymes such as Uge5 (UDP-glucose 4-epimerase), GlfA (UDP-galactopyranose mutase), GlfB (UDP-galf transporter), PmtA (O-mannose type mannosyltransferase), Mnt1 (mannosyltransferase), Gfts (hypothetical galactofuranosyltransferase), GfsA/C (galactofuranosyltransferase), along with key substrates Glu (glucose), UDP-Gal*p* (UDP-galactopyranose), UDP-Gal*f* (UDP-galactofuranose), and Man (mannose).

Integrated analysis of acid stress-induced metabolic shifts reveals the regulatory network in Figure 9. At pH 5.0, impaired galactomannan synthesis in mutants promotes MP efflux. Conversely, pH 3.0 upregulates specific wall polysaccharide genes to bolster stress resilience. Transcriptional regulation of this process likely involves global regulators ROX1 and SPT15 (Figure 8). Environmental signals stimulating the cell wall and membrane activate ROX1 while repressing SPT15. This dual action downregulates growth-associated metabolic pathways including carbon metabolism (propionate, starch/sucrose, glycolysis/gluconeogenesis, steroid biosynthesis, glyoxylate/dicarboxylate, and fatty acid degradation), amino acid metabolism (branched-chain degradation, arginine/proline, tryptophan, galactose, and tyrosine), cofactor biosynthesis, glutathione metabolism, and glycerolipid metabolism. Consequently, reduced growth rates coincide with diminished precursor supply (e.g., coenzyme A), suppressing MP synthesis. Simultaneously, metabolic shifts toward stress adaptation pathways occur. These encompass glycerolipid metabolism, fatty acid biosynthesis, non-homologous end joining, terpenoid–quinone biosynthesis, thiamine metabolism, glycine/serine/threonine metabolism, and cofactor biosynthesis. Such redirection promotes synthesis of resilience-associated compounds (fatty acids, terpenoids, and thiamine), sustaining cellular viability (Figure 3).

Interestingly, acid stress significantly altered glucan synthase expression (*ags1*/*ags3* upregulated, *ags2* downregulated; Figure 4), indicating multi-polysaccharide restructuring in cell wall adaptation. How these structural changes directly or indirectly modulate MP synthesis/secretion requires further investigation.

## 5. Conclusions

Transcriptomic profiling of wild-type (M9-WT) and knockout (M9-KO) strains under varying pH conditions (pH 5.0 and 3.0) revealed mechanistic insights into MP biosynthesis under combined cell wall restructuring and acid stress. Integrated analysis encompassing GO/KEGG enrichment, pH-based polysaccharide/pigment synthesis gene correlations, series test of cluster, and transcription factor identification demonstrated that basal pH conditions (pH 5.0) elicited minimal inter-strain transcriptional divergence. This aligns with prior findings wherein *MpglfA* deletion enhanced MP synthesis/secretion via altered cell wall polysaccharide architecture, primarily involving differential expression of primary metabolism genes (k5_w5 comparison). Acid stress (pH 3.0) substantially increased differentially expressed genes across all comparisons. M9-KO exhibited heightened responsiveness versus M9-WT, indicating compromised stress resilience concomitant with cell wall alterations. Metabolic restructuring in M9-KO (k3_k5) favored amino acid and fatty acid degradation pathways that generate MP precursors (acetyl-CoA/propionyl-CoA), suggesting accelerated transition from growth to secondary metabolism. Concurrently, intensified inter-strain differences (k3_w3) reflected robust stress adaptation mechanisms involving activation of osmolyte synthesis pathways coupled with suppression of growth-associated processes.

Association analysis further delineated pH-dependent regulation. Galactomannan synthase genes showed negligible pH responsiveness across conditions, although greater perturbation sensitivity occurred in M9-KO, particularly during inter-strain acid exposure (k3_w3). Glucan synthases demonstrated significantly higher acid sensitivity than galactomannan-related genes. While most MP biosynthetic genes were modestly upregulated at pH 5.0, severe transcriptional repression occurred in M9-KO under acidity. The series test of cluster confirmed primary metabolic pathways, particularly amino acid and carbon metabolism, as critical for MP synthesis. Putative antagonistic regulators ROX1 and SPT15 were implicated in the pH response, although their precise roles in signal transduction from cell wall perturbations and specific pigment regulation require further validation.

## Data Availability

The original contributions presented in this study are included in the article/supplementary material. Further inquiries can be directed to the corresponding authors.

## Supplementary Materials

The following supporting information can be downloaded at https://www.mdpi.com/article/10.3390/foods14213602/s1: Table S1: Statistical table for quality evaluation of sequencing Data; Table S2: Statistics of comparison results between sequencing sequences and reference genomes; Table S3: Statistical analysis of overall gene expression levels in the sample population; Figure S1: Distribution of differentially expressed genes in the pathway of valine, leucine, and isoleucine degradation in subcluster_8; Table S4: The differentially expressed genes and their functions involved in subcluster_5 and subcluster_8; Table S5: Predicted transcriptional regulatory factors and their functions.
