# Supplementary material for: Cell Wall Remodeling and pH Stress Coordinately Regulate Monascus Pigment Biosynthesis Through Transcriptional Reprogramming
Source: Foods. 2025 Oct 23;14(21):3602. doi: 10.3390/foods14213602 (PMC12607504; doi:10.3390/foods14213602)
Supplement: Supplementary file 1 [file foods-14-03602-s001.zip › foods-3893669-supplementary.pdf]

Supplementary Materials

Table S1: Statistical table for quality evaluation of sequencing Data;
Table S2: Statistics of comparison results between sequencing sequences and reference genomes;
Table S3: Statistical analysis of overall gene expression levels in the sample population;
Figure S1: Distribution of differentially expressed genes in the pathway of valine, leucine, and isoleucine degradation in subcluster_8;
Table S4: The differentially expressed genes and their functions involved in subcluster_5 and subcluster_8;
Table S5: Predicted transcriptional regulatory factors and their functions.

Table S1. Statistical table for quality evaluation of sequencing Data.

Samples	Raw reads	Raw bases (Gb)	Clean reads	Clean bases (Gb)	Error rate (%)	Q20 (%)	Q30 (%)	GC Content (%)
w5_3	44728690	67.54	44278714	65.61	0.0271	97.22	92.27	53.57
w5_2	43543510	65.75	43219220	63.71	0.0264	97.52	92.92	53.33
w5_1	62544874	94.44	61948928	90.21	0.0265	97.51	92.94	53.33
k5_3	44657632	67.43	44064862	65.01	0.0274	97.10	92.01	54.39
k5_2	54166798	81.79	53660180	78.50	0.0266	97.48	92.81	53.11
k5_1	45366684	68.50	44813816	66.18	0.0269	97.30	92.44	53.88
w3_3	46438764	70.12	45980486	67.83	0.0265	97.46	92.82	53.17
w3_2	57422676	86.71	56804730	83.48	0.0273	97.17	92.11	53.11
w3_1	44841248	67.71	44524058	65.31	0.0261	97.64	93.17	53.05
k3_3	50781478	76.68	50296618	74.33	0.0274	97.13	92.03	53.18
k3_2	43675386	65.95	43270362	64.25	0.0270	97.28	92.40	53.49

Overall Alignment Rate (%) | Multimapping Rate (%) | Uniquely Mapped Rate (%)

Table S2. Statistics of comparison results between sequencing sequences and reference genomes.

Samples	Total reads	Total alignment rate (%)	Multimapping Rate (%)	Uniquely Mapped Rate (%)
w5_3	44278714	41119592(92.87%)	293457(0.66%)	40826135(92.2%)
w5_2	43219220	39733788(91.94%)	318379(0.74%)	39415409(91.2%)
w5_1	61948928	57688096(93.12%)	396395(0.64%)	57291701(92.48%)
k5_3	44064862	41773049(94.8%)	294584(0.67%)	41478465(94.13%)
k5_2	53660180	50178825(93.51%)	357265(0.67%)	49821560(92.85%)
k5_1	44813816	42474150(94.78%)	295504(0.66%)	42178646(94.12%)

w3_3	45980486	42946339(93.4%)	297097(0.65%)	42649242(92.76%)
w3_2	56804730	52999426(93.3%)	367277(0.65%)	52632149(92.65%)
w3_1	44524058	41692750(93.64%)	286325(0.64%)	41406425(93.0%)
k3_3	50296618	47444644(94.33%)	423465(0.84%)	47021179(93.49%)
k3_2	43270362	40764500(94.21%)	287131(0.66%)	40477369(93.55%)
k3_1	53159270	49992893(94.04%)	384312(0.72%)	49608581(93.32%)

Table S3. Statistical analysis of overall gene expression levels in the sample population.

samples	FPKM range					
	0-1	1-3	3-10	10-50	> 50	total
w5_3	150(3.25%)	177(3.83%)	456(9.87%)	1663(36%)	2173(47.04%)	4619(100%)
w5_2	189(4.09%)	205(4.44%)	477(10.33%)	1668(36.11%)	2080(45.03%)	4619(100%)
w5_1	124(2.68%)	190(4.11%)	436(9.44%)	1610(34.86%)	2259(48.91%)	4619(100%)
k5_3	95(2.06%)	228(4.94%)	645(13.96%)	1703(36.87%)	1948(42.17%)	4619(100%)
k5_2	53(1.15%)	160(3.46%)	429(9.29%)	1683(36.44%)	2294(49.66%)	4619(100%)
k5_1	203(4.39%)	206(4.46%)	527(11.41%)	1569(33.97%)	2114(45.77%)	4619(100%)
w3_3	52(1.13%)	129(2.79%)	400(8.66%)	2420(52.39%)	1618(35.03%)	4619(100%)
w3_2	40(0.87%)	113(2.45%)	420(9.09%)	2419(52.37%)	1627(35.22%)	4619(100%)
w3_1	38(0.82%)	114(2.47%)	416(9.01%)	2437(52.76%)	1614(34.94%)	4619(100%)
k3_3	101(2.19%)	159(3.44%)	401(8.68%)	1540(33.34%)	2418(52.35%)	4619(100%)
k3_2	126(2.73%)	180(3.9%)	443(9.59%)	1562(33.82%)	2308(49.97%)	4619(100%)
k3_1	124(2.68%)	184(3.98%)	411(8.9%)	1570(33.99%)	2330(50.44%)	4619(100%)

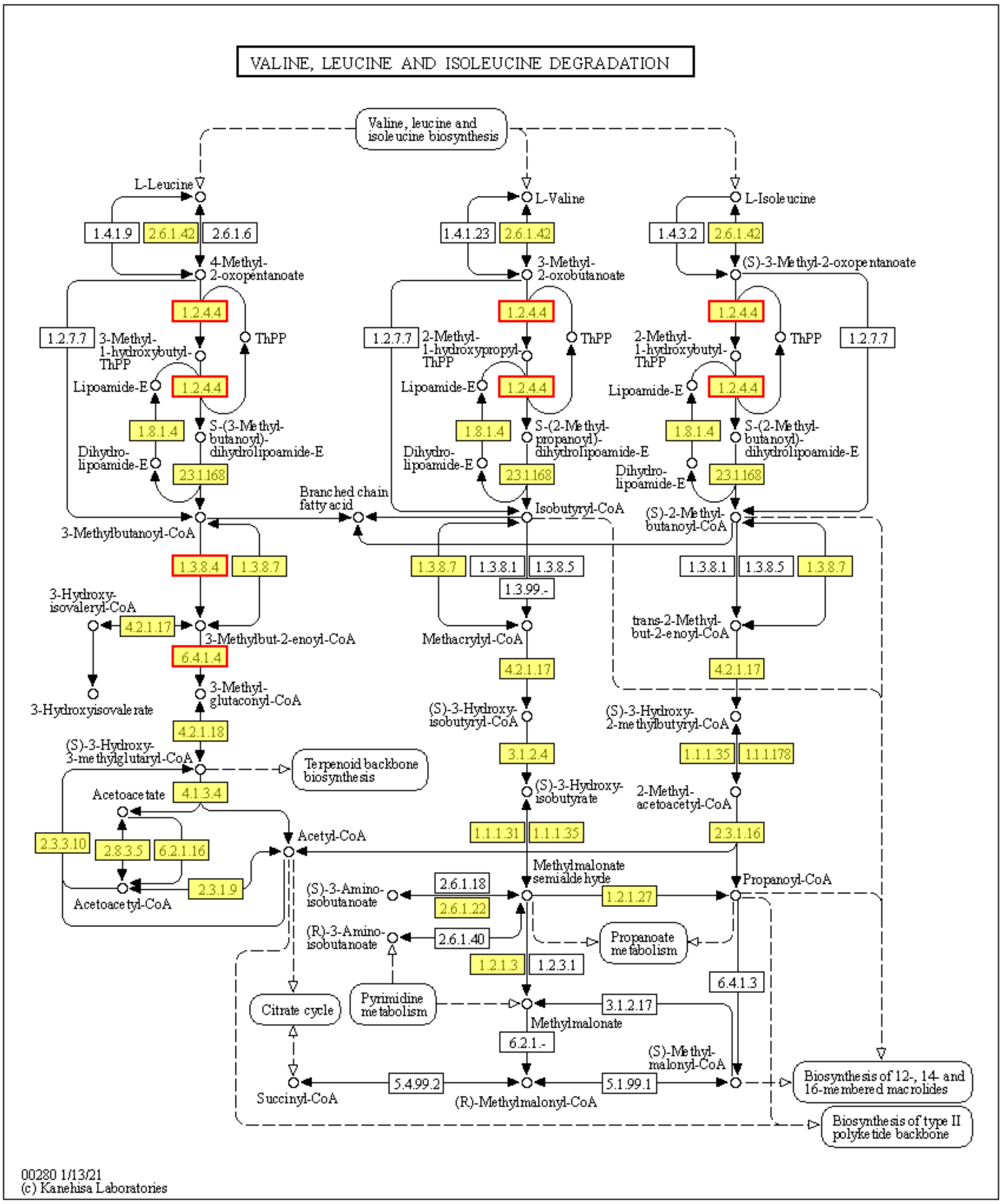

Figure S1. Distribution of differentially expressed genes in the pathway of valine, leucine, and isoleucine degradation in subcluster_8. Note enzyme 1.2.4.4, 1.3.8.4 and 6.4.1.4 were coded by gene-MPDQ_004788, gene-MPDQ_003394 and gene-MPDQ_003395 in *Monascus*, respectively.

Table S4. The differentially expressed genes and their functions involved in subcluster_5 and subcluster_8.

Subcluster	Gene ID	Gene Name	Gene Function
subclus	gene-	ATP1	Mitochondrial ATP synthase F1 sector α subunit

ter_5	MPDQ_002787		
	gene-MPDQ_004619	FUM1	Fumarase fum1
	gene-MPDQ_005516	SDH4	Succinate dehydrogenase membrane anchor subunit Sdh4
	gene-MPDQ_002295	SHM2	Serine hydroxymethyltransferase (cytosolic)
	gene-MPDQ_004892	CYC1_1	Iso-1-cytochrome c
	gene-MPDQ_004743	COX15	Cytochrome c oxidase assembly protein cox15
	gene-MPDQ_004783	ARG1	Argininosuccinate synthase
	gene-MPDQ_001991	QCR7	Cytochrome b-c1 complex subunit 7
	gene-MPDQ_002529	MPDQ_002529	Hypothetical protein
	gene-MPDQ_005570	ATP20	ATP synthase subunit G, mitochondrial
	gene-MPDQ_000201	ADE13	Adenylosuccinate lyase ade13
	gene-MPDQ_004220	ATP9	ATP synthase complex subunit 9
	gene-MPDQ_002423	MPDQ_002423	Hypothetical protein
	gene-MPDQ_007043	COX5	Cytochrome c oxidase subunit 5
	gene-MPDQ_000341	ILV5	Bifunctional acetohydroxy acid reductoisomerase
	gene-MPDQ_005981	ADE17	Bifunctional phosphoribosylaminoimidazolecarboxamide formyltransferase/IMP cyclohydrolase
	gene-MPDQ_000708	UGA1	4-aminobutyrate transaminase
	gene-MPDQ_000336	MPDQ_000336	Hypothetical protein
	gene-MPDQ_001959	QCR2	Ubiquinol-cytochrome c reductase core subunit 1
	gene-MPDQ_001262	KGD2	2-oxoglutarate dehydrogenase complex E2 component
	gene-MPDQ_005976	LYS4	Mitochondrial homoaconitase
	gene-MPDQ_003443	MPDQ_003443	Hypothetical protein

	gene-MPDQ_002058	SHM1	Glycine hydroxymethyltransferase shm1
	gene-MPDQ_000697	CPA1	Multifunctional pyrimidine synthesis protein CAD
	gene-MPDQ_004009	MPDQ_004009	Hypothetical protein
	gene-MPDQ_003961	GCV2	Glycine decarboxylase subunit P
	gene-MPDQ_001921	MPDQ_001921	Hypothetical protein
	gene-MPDQ_007389	IDH1	Isocitrate dehydrogenase (NAD(+)) idh1
	gene-MPDQ_005224	MPDQ_005224	Hypothetical protein
	gene-MPDQ_001519	ASN1	Asparagine synthase
	gene-MPDQ_004020	GCV1	Aminomethyltransferase, mitochondrial
	gene-MPDQ_002124	MPDQ_002124	Hypothetical protein
	gene-MPDQ_003841	MPDQ_003841	Hypothetical protein
	gene-MPDQ_006647	ACO2	Aconitate hydratase
	gene-MPDQ_008111	ILV3	Dihydroxy acid dehydratase ilv3
	gene-MPDQ_007362	MPDQ_007362	Hypothetical protein
	gene-MPDQ_007050	MPDQ_007050	Hypothetical protein
	gene-MPDQ_002218	HOM3	Aspartate kinase
	gene-MPDQ_007461	COX6	Cytochrome c oxidase subunit 6
subcluster_8	gene-MPDQ_002999	MPDQ_002999	Hypothetical protein
	gene-MPDQ_003584	MPDQ_003584	Hypothetical protein
	gene-MPDQ_003395	MPDQ_003395	Hypothetical protein
	gene-MPDQ_005940	CAR2	Ornithine aminotransferase
	gene-MPDQ_004788	MPDQ_004788	Hypothetical protein

gene-MPDQ_003394	MPDQ_003394	Hypothetical protein

Table S5. Predicted transcriptional regulatory factors and their functions.

Gene names	Factors	Function (swiss prot annotation)
MPDQ_008202	SPT15	TATA-box-binding protein
MPDQ_005744	GZF3	GATA type zinc finger protein
MPDQ_004573	GAT1	Nitrogen regulatory protein areA
MPDQ_004250	RLM1	MADS-box MEF2 type transcription factor MIG1
MPDQ_005936	MSN-1	C2H2 finger domain transcription factor sebA
MPDQ_000960	CRE-1	Probable DNA-binding protein creA
MPDQ_002140	RST2	Transcriptional regulator ADR1
MPDQ_002084	SFP1	Transcription factor SFP1
MPDQ_005046	RFX1	Protein sak1
MPDQ_007404	ROX1	Transcription factor rox1
MPDQ_000494	YOX1	Homeobox protein YOX1
MPDQ_004589	MCM1	Transcription factor of morphogenesis MCM1
MPDQ_005349	HAP2	Transcriptional activator HAP2
MPDQ_006319	CAT8	Transcriptional activator protein acu-15
